# Supplementary material for: Genome-resolved correlation mapping links microbial community structure to metabolic interactions driving methane production from wastewater
Source: Nat Commun. 2023 Sep 4;14:5380. doi: 10.1038/s41467-023-40907-5 (PMC10477309; doi:10.1038/s41467-023-40907-5)
Supplement: Supplementary file 2 — Description of Additional Supplementary Files [file 41467_2023_40907_MOESM2_ESM.pdf]

Description of Additional Supplementary Files:

Supplementary Data 1: Inventory table of all samples collected for 16S rRNA sequencing and metagenomics + metatranscriptomics ("omics"). Date collected and all physicochemical parameters with units are shown in columns.

Supplementary Data 2: Accounting table for metatranscriptome sequencing and read mapping to metagenome contigs and bins.

Supplementary Data 3: Accounting table for metagenome sequencing, assembly, binning, and read mapping to contigs. Database deposition and accession numbers are listed for each metagenome.

Supplementary Data 4: Statistics table of two-sided PERMANOVA tests for pairwise comparisons of physiochemical variable profiles between process configurations.

Supplementary Data 5: Summary table of ASV taxonomy and observed counts across the time series.

Supplementary Data 6: Statistics table of two-sided PERMANOVA tests for pairwise comparisons of taxonomic (ASV) abundance profiles between process configurations.

Supplementary Data 7: Summary table of the bio.env test to determine the combination of physiochemical variables with the highest explanatory power for ASV profiles.

Supplementary Data 8: Summary table of the ASV node information from the parent network. Mean_Rel_Abund is the average relative abundance of the ASV across the time series, Indicator_Configuration is the process configuration in which an ASV was identified by multi-level pattern analysis as an indicator species, Node_MAG shows ASV-MAG pairs which appear in the parent network, and Subnetwork shows which methanogen-seeded subnetwork each ASV belongs to (if any).

Supplementary Data 9: Summary table of all edges in the parent network. Bicor is the correlation score of two ASVs (Node1 and Node2) and p-value is a false discovery rate corrected p-value of the Bicor test.

Supplementary Data 10: Summary table of sequence information and taxonomy for all medium-quality (MQ) and high-quality (HQ) MAGs identified in the study. The Metagenome_TPM and Metatranscriptome_TPM are summed genomic and transcriptomic abundances for each MAG calculated as described in the methods, Sample_Binned shows which metagenome sample (timepoint) a MAG was binned from, Completeness and Contamination scores were determined by CheckM as described in the methods, the Total_Bases and Total_Contigs show size of the bin, and 5S, 16S, and 23S show the count of each gene on contigs in each bin.

Supplementary Data 11: Summary table of functional and taxonomic annotations for all open reading frames encoded by MAGs discussed from the methanogenic subnetworks. Functional database annotations to COG, EC, Pfam, RefSeq, and KO identifiers are described in the methods. The MAG quality, completeness, contamination, and taxonomy are also provided and match TableS10. The metagenome and metatranscriptome abundances (TPM) are provided for each open reading frame.
