# Supplementary material for: Genome-resolved correlation mapping links microbial community structure to metabolic interactions driving methane production from wastewater
Source: Nat Commun. 2023 Sep 4;14:5380. doi: 10.1038/s41467-023-40907-5 (PMC10477309; doi:10.1038/s41467-023-40907-5)
Supplement: Supplementary file 16 — Supplementary Figures [file 41467_2023_40907_MOESM16_ESM.pdf]

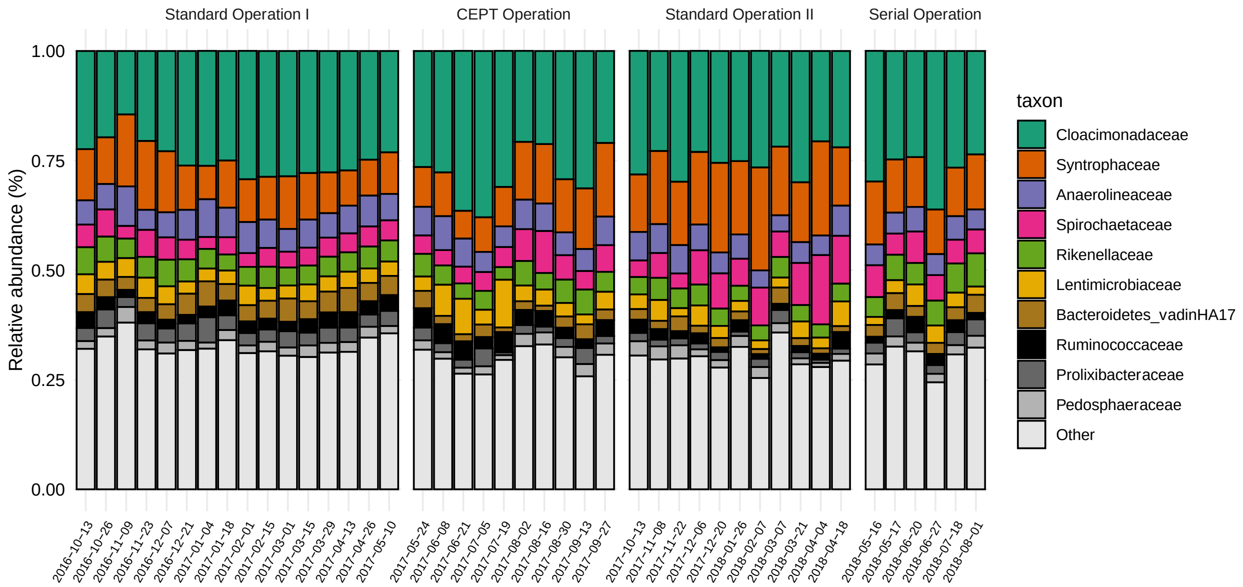

Fig. S1. AD microbial community composition at family-level taxonomic groupings for all sampling dates along the time-series. Replicate samples for each date are averaged, and the relative abundances of all ASVs within a given family are summed.

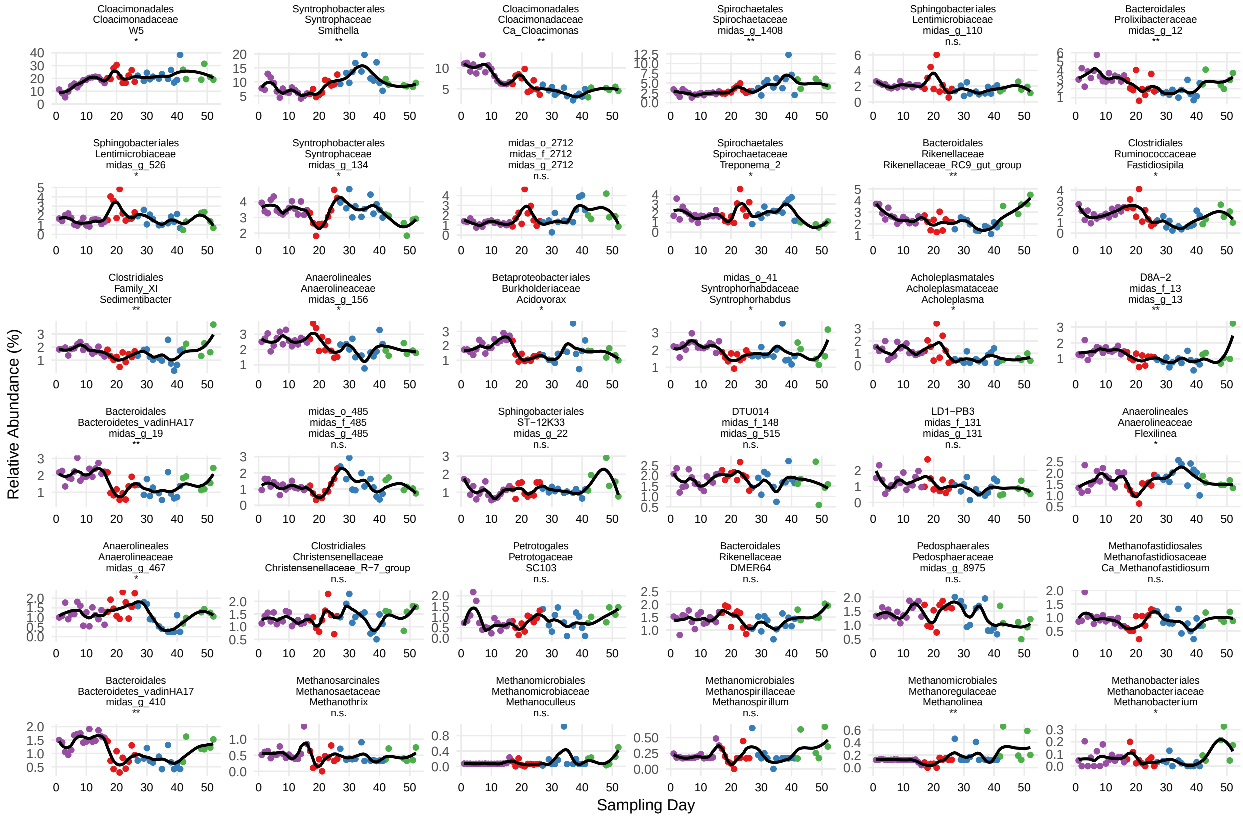

Fig. S2. The relative abundances of genus-level taxa across the time-series. All ASVs within each genus are summed. The y-axis represents sample number (1-52) – date and sample name information can be found in Supplemental Table S1. Points are samples colored by configuration (violet = SOI, red = CEPT, blue = SOII, green = SER). Each graph facet includes the order, family, and genus name, and whether a two-sided ANOVA test determined that the genus had differential abundance between configurations (n.s. = not significant, * = p<0.05, ** = p < 0.01, *** = p < 0.001).

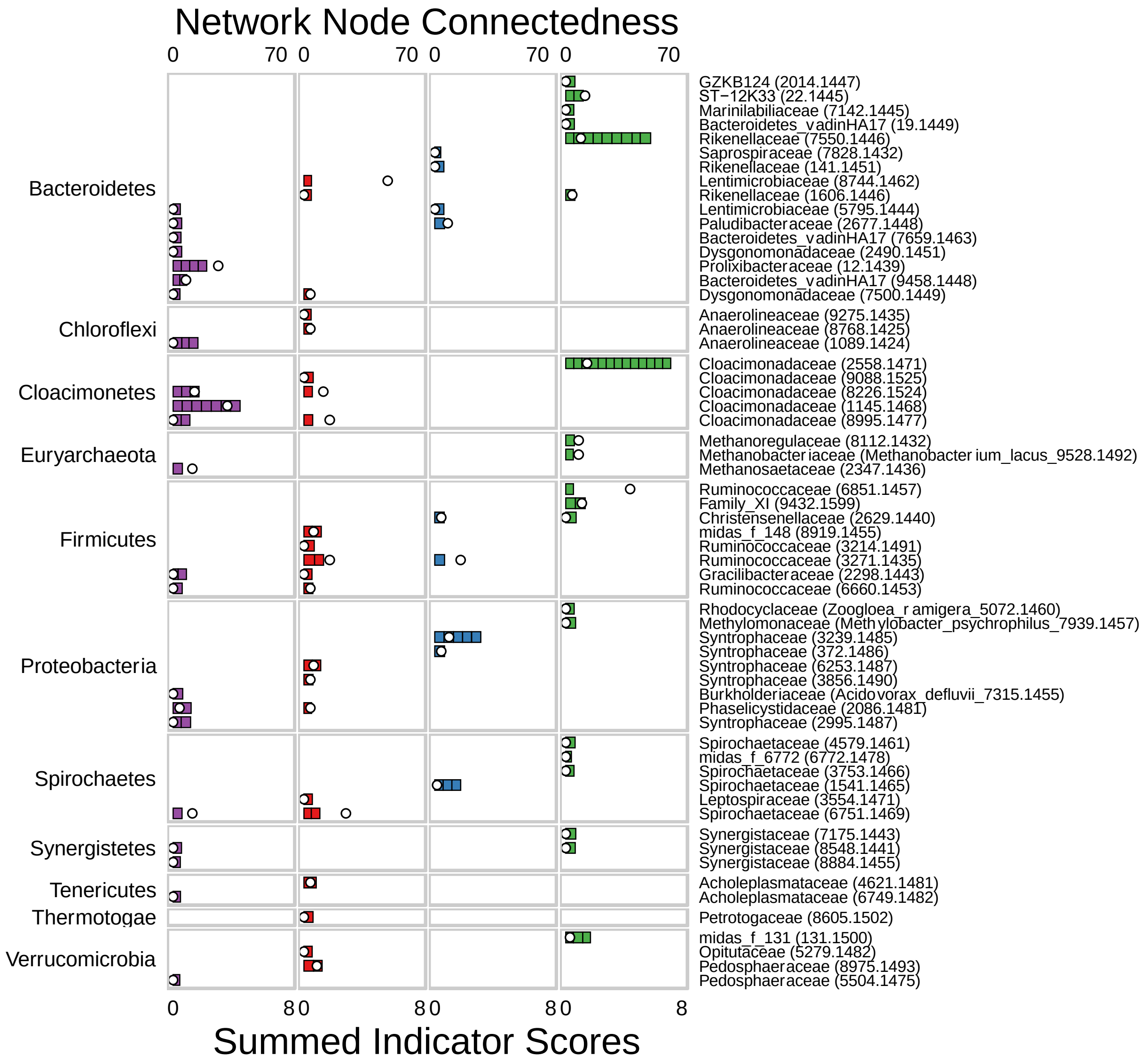

Fig. S3. Indicator ASVs are shown as individual boxes, faceted by the configuration that they are indicators for (left to right: violet = SOI, red = CEPT, blue = SOII, green = SER). Each ASV box is in a row which indicates its family-level taxonomy (and MIDAS ID) on the right axis and its phylum-level taxonomy on the left axis. The bottom x-axis shows the summed and individual multi-pattern-level analysis score for indicator ASVs and the top x-axis shows the summed network connectedness of indicator ASVs from each family (see Methods and Figure 4).

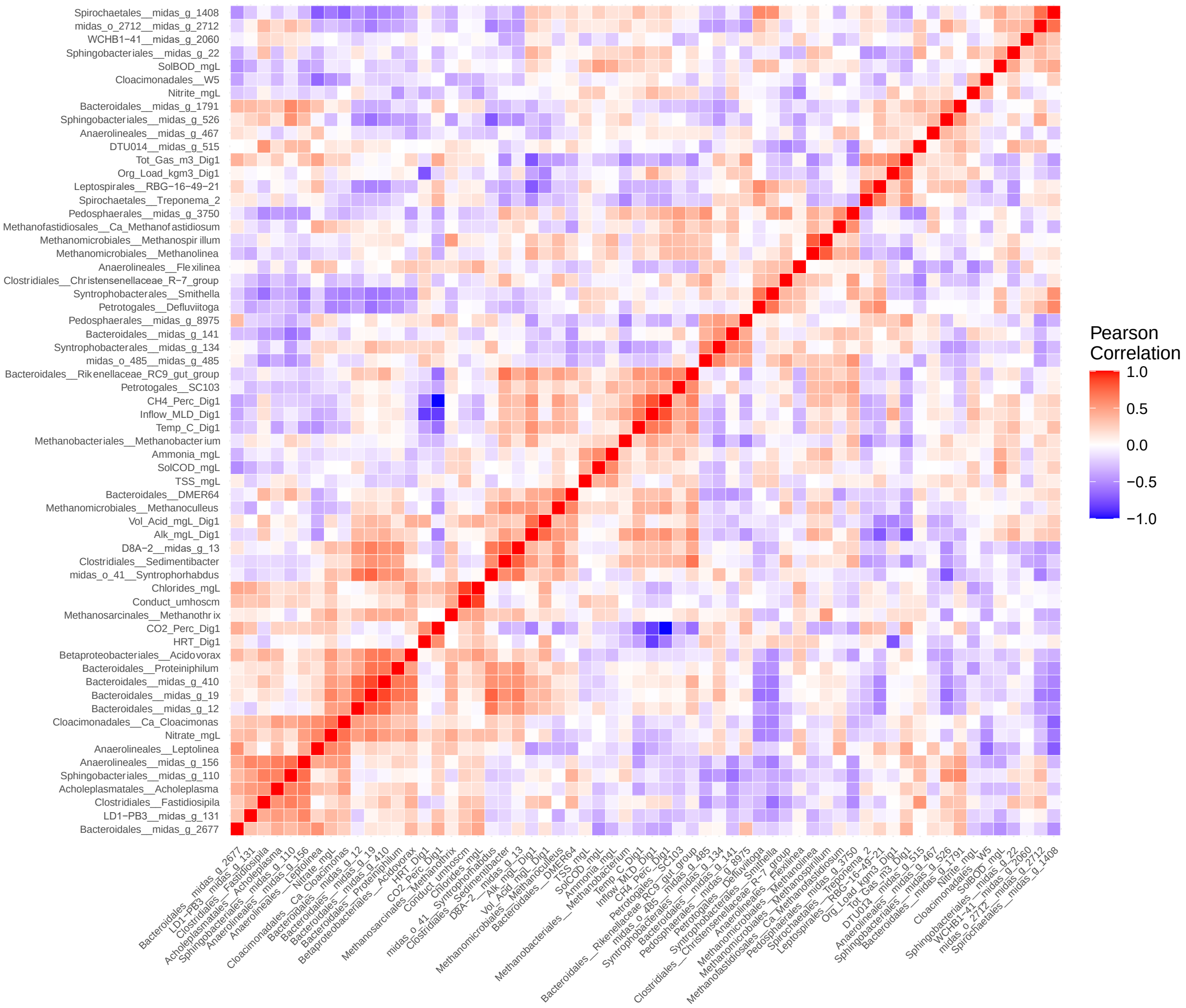

Fig. S4. Pearson correlation across the time-series samples of the relative abundance of the dominant genus-level taxa in the AD and the physicochemical parameters measured from AD digestate. Rows and columns are organized by a Bray-Curtis dissimilarity dendrogram of Pearson rho values.

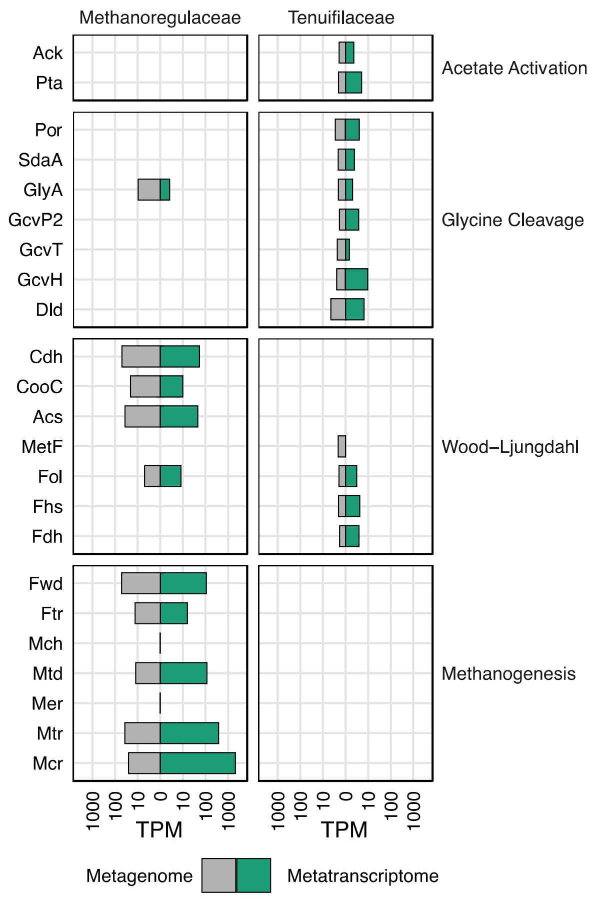

Fig. S5. TPM values from metagenomic and metatranscriptomic read mapping to select genes in the Subnetwork 1 MAGs from Methanoregulaceae (3300036947_39) and Tenuifilaceae (3300028576_27). TPM values and other genomic features are available in Table S11.
